# Supplementary material for: Triple-Negative Breast Cancer Histological Subtypes with a Favourable Prognosis
Source: Cancers (Basel). 2021 Nov 14;13(22):5694. doi: 10.3390/cancers13225694 (PMC8616217; doi:10.3390/cancers13225694)
Supplement: Supplementary file 1 [file cancers-13-05694-s001.zip › cancers-1445792-supplementary.pdf]

Supplementary Tables S1–S6

Table S1. Summary of reported cases of acinic cell carcinomas.

nr.	References First Author	sex/age	tumor size (mm)	pT	node status	surgery	adjuvant therapy	follow up (months)	recur-rences/metas-tases	growth pattern
**1**	Roncaroli [16]	F/42	30	T2	1/18	MRM + ALDN	CT	60	-	solid
**2**	Shimao [17]	M/23	48	T2	ns	BCS + ALDN	-	34	-	solid
**3**	Damiani [18]	F/35	40	T2	2/20	MRM + ALDN	Neo-CT	12	-	microglandular
**4**		F/63	50	T2	ns	BCS	-	48	local	microglandular
**5**		F/64	33	T2	0/8	BCS + ALND	-	12	-	microglandular
**6**		F/80	20	T2	ns	BCS	HT	12	-	microglandular
**7**	Schmitt [19]	F/79	45	T2	0/23	MRM + ALDN	RT	21	-	solid
**8**	Coyne [20]	F/49	20	T1c	2/11	MRM + ALDN	Neo-CT + Ad-CT	36	liver	microglandular
**9**	Hirokawa [21]	F/20	25	T2	0	MRM + ALDN	-	6	-	papillary-cystic
**10**		F/61	25	T2	0	MRM + ALDN	-	24	-	papillary-cystic, microcystic, follicular
**11**		F/59	10	T1b	0	MRM + ALDN	-	84	-	microfollicular
**12**	Kahn [22]	F/56	22	T2	0/18	MRM + ALDN	-	28	-	solid
**13**	Peintinger [23]	F/36	35	T2	0/15	BCS + ALDN	CT + RT	120	lung	solid and microglandular
**14**	Tanahashi [24]	F/80	21	T2	0/1	MRM + SLDN	-	22	-	acinar
**15**	Huo [25]	F/40	36	T2	1/21	MRM + ALDN	Neo-CT + RT + HT	12	-	microglandular, solid, focally microcystic
**16**		F/30	26	T2	2/33	BCS + ALDN	CT + RT	34-died	bone met.	microglandular
**17**	Choh [26]	F/79	27	T2	0/1	BCS + SLND	RT	9	-	ns
**18**	Sakuma [27]	F/61	14	T1c	0	BCS + ALDN	-	14	-	solid, microcystic
**19**	Zhao [28]	F/38	30	T2	0/23	MRM + ALDN	CT	10	-	microglandular
**20**	Shingu [29]	F/41	35	T2	0/1	BCS + SLND	CT + RT	36	-	solid, trabecular, microglandular
**21**	Osako [30]	F/50	52	T3	0/30	MRM + ALDN	-	184.8	-	microglandular
**22**		F/37	22	T2	0/23	BCS + ALDN	-	139.2	-	microglandular, solid
**23**		F/46	50	T2	0/4	MRM + SLDN	-	64.8	-	papillary, microglandular, solid, cystic
**24**	Winkler [31]	F/56	47	T2	0/1	MRM + SLDN	Neo-CT + HT	24	-	solid

**25**	Ripamonti [32]	F/44	13	T1c	0/2	MRM + SLDN	HT	19	-	microglandular
**26**	Limite [33]	F/26	16	T1c	0/1	BCS + SLND	-	8	-	acinic-glandular, solid
**27**	Guerini-Rocco [34]	F/36	50	T2	10/17	ns	ns	24-died	ns	clear cell
**28**		F/55	19	T1c	0/8	ns	ns	132	-	microglandular
**29**		F/34	36	T2	0/3	ns	ns	15	-	microglandular
**30**		F/48	20	T1c	ns	ns	ns	60	-	microglandular
**31**		F/35	18	T1c	2/22	ns	ns	72	local	microglandular
**32**	Conlon [35]	F/47	23	T2	1/18	MRM + ALDN	CT + RT + HT	72	local	microglandular, solid
**33**		F/49	11	T1c	0	MRM + SLDN	Neo-CT	18	-	microglandular, solid
**34**	Li [36]	F/52	15	T1c	0/1	Simple Mx	-	3	-	Microglandular cystic
**35**	Sen [37]	F/41	25	T2	0/1	MRM	CT+RT	12	-	Microglandular, solid

F= female; M= Male; R= Right; L: Left; T = tumour; CT= chemotherapy; ns, not stated; RT, radiotherapy; uiq, upper internal quadrant; uoq, upper outer quadrant, Mx: mastectomy, MRM: modified radical mastectomy, BCS: breast conserving surgery

Table S2. Literature review of adenoid cystic carcinoma of the breast focusing on classical adenoid cystic carcinoma (CAdCC) with data on treatment and outcome.

Reference	Number of cases (with grade)	Age	Size (mm)/presentation	Surgical treatment	LN status	Immunophenotype	Adjuvant treatment	Outcome {FU}
Elimimian [48]<br>NCDB 2010-2016	1357<br>Grade 1: 520<br>Grade 2: 320<br>Grade 3-4: 145	62 (median)	not detailed	not details	3.6% pos (39/1088)	ER+ 25.3%	RT 52.4%<br>CT 12.9%<br>ET 11.2%	88.4% (5-year-OS) for all grades {mean 55.6 mo}
Kulkarni [49]<br>NCDB 1998-2008	933<br>Grade 1: 429<br>Grade 2: 337<br>Grade 3: 167	60 (median)	18 (median)	BCS 69.8%	5.1% pos.	391 evaluated:<br>ER+ 15.4%<br>PR+ 13.3%<br>HER-2 NA	RT 47.1%<br>CT 11.3%<br>ET 8.9%	88% (OS) {66 mo; median}; 91% (OS) for Grade 1 AdCC
Li [50]<br>SEER	696<br>Grade Low: 272<br>High: 67	61,5 (mean)	22 (mean)	S 67.7%	4,5% pos.	NA	S+RT 30.4%<br>RT only 0.3%; no SEER data on CT	96.8% (DSS Loc);<br>78.5% (DSS regional)<br>15.9% (DSS distant)<br>{NA}
Khanfir [51]<br>Rare Cancer Network	61<br>Grade (Bloom-Richardson)<br>G1: 10<br>G2: 1<br>G3: 1<br>Gx: 49	59 (median)	20 (median)	BCS 67%	neg	In 45 pts evaluated:<br>ER+ 7%<br>PR+ 5%<br>HER-2- 100%	RT 66%<br>CT 13%	94% (OS)<br>82% (DFS)<br>{79 mo; median}
Thompson [52]<br>California Tumor Registry	244<br>Grade NA	61.9 (median)	37 (mean for LN+)<br>22 (mean for LN-)	NA	4.9% pos.		NA	95.5% (RCS)<br>{NA}

Coates [53]<br>SEER database	376<br>Grade NA	62<br>(mean)	NA	BCS 60%	6.5% pos.	NA	RT 52% no SEER data on CT	NA
Ghabach [46]<br>SEER-9 database	338<br>Grade NA	62 (me-dian)	18 (median)	NA	2% pos	ER+ 12%<br>PR+ 8%<br>HER-2 NA	RT 35% no SEER data on CT	98,1 %(OS)<br>{30 y}
Arpino [54]	28<br>Grade NA	66 (me-dian)	19 (median)	Mx 79%<br>BCS 21%	4% pos	ER+ 46%<br>PR+ 36%<br>HER-2 NA	RT 21%<br>CT 4%<br>ET 26%	100% (5 y DFS)<br>85% (OS)<br>{83 mo; median}
Treitl [55]	6<br>Grade NA	66 (me-dian)	16 (mean)	S 100%	neg	ER+ 20%<br>PR+ 0%<br>HER-2- 67%, NA 33%	RT 17%<br>ET 17%	100%<br>{85 mo; median}
Zhang [56]	14<br>Grade 1: 9<br>Grade 2: 3<br>Grade 3: 1	60.5 (me-dian)	PM 85.7%	Mx+ALND/SLNB:29%/43%<br>BCS+SLNB: 21%<br>WLE: 7%	neg	ER+ 43%<br>PR+ 14%<br>Her-2- 100%	RT 29%<br>CT 14%<br>ET 36%	85.7% (5yDFS)<br>{59 mo; median}<br>Relapses in 2 HR+ cases with perineu-rial invasion
Kim [57]	6<br>NG1: 2/6<br>NG2: 1/6 case<br>NG3: 1/6<br>NGx: 2/6	52<br>(mean)	PM 83%	BCS + SLNB: 33%<br>BCS+ALND: 17%<br>Mx+SLNB: 17%<br>Mx +ALNB:33%	neg	ER- 100%<br>PR- 100%<br>HER-2- 100%	RT 33%<br><br>CT 66%	OS not calculated<br>Metastases in 2 cases:<br>case NG-1 (lung)<br>case NG-3 (lung, bone & liver)<br>{NA}
Millar [58]	19<br>Grade 1: 2/7<br>Grade 2: 4/7<br>Grade 3: 3/7	58 (me-dian)	22 (median)<br>PM 84%	Lu: 16%<br>Mx: 5%<br>Lu+LN: 37%<br>Mx+LN: 42%	27% pos	NA	RT 47%<br>CT 16%<br>ET 5%	88% (OS)<br>{168 mo; median}<br>Recurrence in breast: 33%

Defaud-Henon [59]	30<br>Grade NA	61 (me-dian)	PM 95%	Lu: 80%<br>Mx: 8.7%	neg	NA	RT 4.3%<br>CT 8.7%	87% (OS)<br>{84 mo; median}<br>local recurrences: 3/23<br>distant metastases (bone, liver, lung): 3/23
Franzese [60]	13<br>Grade 1: 9/13<br>Grade 2: 4/13	51 (me-dian)	PM 92%	Lu: 100%<br>ALND: 85%<br>SLNB: 15%	15% pos	ER+ 31%<br>PR+ 31%<br>HER-2+ 8%	RT 100%<br>CT 15%<br>ET 23%	100% (OS)<br>{74 mo; median}<br>Breast recurrence: 1/13<br>Distant metastasis (lung): 1/13
Yigit [61]	7<br>Grade 1: 5/7<br>Grade 2: 2/7	58 (me-dian)	PM 86%	Mx: 43%<br>BCS: 57%<br>ALND:71%<br>SLNB: 14%	neg	ER+ 14%<br>HER2+ 0%<br>AR+ 86%	RT: 57%<br>CT: 86%<br>ET: 29%	86% (OS)<br>{86 mo; median}<br>Recurrence: 2/7 (both G1)
Marco [62]	17<br>Nottingham grade<br>1: 11<br>2: 3<br>3: 3	59.9 (mean)	26 (mean)	BCS: 76%<br>Mx: 24 %	10% pos	ER- 100%<br>PR- 100%<br>HER2 neg 100%	RT: 46%<br>CT: 8%	{90 mo; mean}<br>Axillary recurrence in 1/17 (high grade transformation)<br>Metastases in 2/17: G3 (lung) and with high grade transfor-mation (lung, colon, brain)
Herzberg [63]	1<br>Grade NA, (cribri-form pattern)	57	15	M+ALND	Neg	NA	No	Distant metastases:<br>-after 6 y lung metas-tasis

								(cribriform pattern),<br>-after 12 y kidney metastasis<br>(cribriform pattern).
Koller [64]	1	76	PM	Mx	Neg	NA	No (CT, RT for relapse after 10 y)	Relapse in breast (at 1 y, 5 y both excised; 10 y – CT/RT only then)<br>Distant metastases: lungs (8.y – not treated), brain (12 y – excised in toto) {lost at 17 y, alive}
Vranic [65]	1<br>Grade NA	76	18	Mx+ALND	Neg	ER, PR, HER-	No	Distant metastasis after 5y<br>-Kidney {5 y}
Silva [66]	1<br>Grade NA	37	30	Mx+ALND	Pos	NA	CT<br>RT	Distant metastases: -brain, liver, lungs, bone {3y}
Mhamdi [67]	1<br>Grade NA	65	80	Mx+ALND	Neg	ER, PR, HER-2 neg	RT	Recurrence in breast after 4 y<br>Distant metastases (after)<br>-lung (5y)<br>-kidney(6y)<br>-brain(7y)
Slodkowska [68]	108 (64 CACC):<br>Nottingham Grade<br>G1: 51<br>G2: 12<br>G3: 1	58 (median)	21 (median)	Lu: 54%<br>Mx: 46%	10% pos<br>In CACC: 0% pos	ER+ 13 %<br>PR+ 3 %<br>HER-2 neg 100%	RT 51%<br>CT 25%	{51 mo; median of 87 pts}<br>CACC G-1: Local recurrence: 8%: ipsilateral axilla, breast

								CACC G-1: Distant metastasis: 8%, mediastinal LN, lung metastases; patient declined treatment and DOD 29 mo

ALND: axillary lymph node dissection, BCS: Breast conserving surgery, CACC: classic ACC (adenoid cystic carcinoma), AR: androgen receptor, CT: chemotherapy, DFS: disease-free survival, DOD: dead of disease, DSS: disease specific survival, ER: oestrogen receptor, ET: endocrine therapy, G: grade, HER2: human epidermal growth factor receptor-2, Loc: Localized, LN: Lymph node, Lu: lumpectomy, mo: .month(s) Mx: mastectomy, NA: not available, neg: negative (-), NG: nuclear grade, OS: overall survival, PM: palpable mass, pos: positive (+), PR: progesterone receptor, pts: patients, RCS: relative cumulative survival, RT: radiation therapy, S: Surgery, SLNB: sentinel lymph node biopsy, WLE: wide local excision, y: year(s).

Table S3. Literature review of fibromatosis-like metaplastic carcinoma (FLMC) of the breast.

Reference (First author)	Pts. #	Age (y)	Presen-tation	Diameter (mm)	LNM at presenta-tion (# of cases)	DM at presenta-tion (# of cases)	Primary diagnosis on CNB/VAB/FNA	Surgical treat-ment	ALND or SLNB (# of cases)	RT (# of cases)	CT (# of cases)	HT (# of cases)	F.U. (mo)	Outcome
Gobbi (1999) [75]	30	63.4 (me-dian) (range: 40–80)	SMP	27 (mean) (12–70 range) (n=28)	0/11 (n=11)	0/11 (n=11)	n. a.	8 x DE 10 x WE 5 x ME (n=23)	11/23 ALND (n=23)	3/23	2/23	1/23	Median: 27 (6–88, range) (n=18)	8/18 LR after median 15.5 mo (range: 5-72 mo) 0/18 DM
Sneige (2001) [76]	24	66.0 (mean) (range: 55-85)	SMP	28 (mean) (range: 12–70) (n=24)	0/18 (n=18)	0/18 (n=18)	n. a.	5 x DE 1 x WE 14 x ME (n=23)	15/22 ALND (n=23)	5/22	0/22	0/22	Median 33 (8–90, range) (n=16)	2/16 LR after 5 and 32 mo 2/16 DM within 24 mo 2/16 DOD (at 17 and 19 mo)
Schafernak (2006) [77]	1	59	SMP	30	n. a.	n. a.	1/1 CNB inconclusive result	1x DE	n. a.	n. a.	n. a.	n. a.	n. a.	n. a.
Rekhi (2007) [78]	1	77	SMP	20	0/1	0/1	1/1 FNA inconclusive result	1x DE	0/1 ALND	1/1	0/1	0/1	n. a.	n. a.
Podetta (2009) [79]	2	72/85	SMP	30 - 57	0/2	0/2	n. a.	1 x WE 1 x ME	2/2 ALND	n. a.	n. a.	n. a.		n. a.
Lamovec (2010) [80]	1	48	SMP	10	0/1	0/1	1/1 FNA Suspicious for ma-lignancy	1 x DE (secondary ME for recur-rence after 46 mo)		1/1	1/1		72	1/1 LR(3x) after 24, 36 and 46 mo 1/1 RLNR after 46 mo 1/1 DM after 64 mo 1/1 DOD at 96 mo
Nonnis (2012) [81]	1	73	SMP	20	0/1	0/1	n. a.	1 x DE	0/1 ALND	0/1	0/1	0/1	16	1/1 LR after 9 mo 0/1 DM
Rito (2014) [82] 1 x EC <5% 1 x SQC <5%	3	Range: 54 – 72	SPM	50 (2 x n. a.)	1/3	0/3	3 /3 CNB FLMCA	2 x WE 1 x ME	3/3 ALND	1/3	1/3 (PCT)	0/3	2 - 17	1/3 LR after 13 mo 1/3 RLNR after 16 mo and 1/3 DM after 17 mo
Takano (2015) [83]	3	n. a.	n. a.	n. a.	n. a.	n. a.	n. a.	n. a.	n. a.	n. a.	n. a.	n. a.	n. a.	n. a.
Pinilla Pagnon (2017) [84]	1	66	MX StM	n. a.	n. a.	n. a.	n. a.	n. a.	n. a.	n. a.	n. a.	n. a.	n. a.	n. a.

Zhu (2017) [85]	1*	28	SPM	70	0/1	0/1	n. a.	1 x DE	0/1	0/1	n. a.	n. a.	n. a.	n.a.
Zhao (2018) [86]	3	51 - 65	SPM	35 - 40	0/3	0/3	n. a.	1 x DE<br>2 x ME	1/3 ALND<br>1/3 SLNB	1/3	3/3	0/3	12 - 49	1/3 LR<br>after 13 mo
Victoor (2020) [87]	1	65	SPM	19	0/1	0/1	1/1 CNB<br>benign	1 x DE	0/1	0/1	0/1	0/1	24	0/1 LR<br>0/1 RLNR<br>0/1 DM
Takatsuka (2021) [88]	1	56	SPM	20	0/1	0/1	1/1 CNB<br>Inconclusive result<br>1/1 VAB<br>Benign<br>1/1<br>2nd CNB<br>Suspicious for FLMCA	1 x ME (be-cause of R1 after DE)	1/1 SLNB	0/1	1/1	0/1	44	0/1 LR<br>0/1 RLNR<br>0/1 DM
Σ	**70**	**28 - 85**	**59/60 SPM<br>1/60 MX StM**	**19 - 70**	**1 / 44**	**0 / 44**		**20/58 DE<br>14/58 WE<br>23/58 ME**	**35 / 56**	**12/55**	**8 / 56**	**1/ 56**		**14/44 LR<br>2/44 RLNR<br>4/44 DM<br>3/44 DOD<br>16/44 any event**

ALND: axillary lymph node dissection; DE: diagnostic excision; CNB: core needle biopsy; CT: chemotherapy; DM: distant metastasis; DOD: death of disease; FLMC: fibromatosis like metaplastic carcinoma; FNA: fine needle aspiration; HT: hormonal therapy; LNM: lymph node metastases; LR: local recurrence ME: mastectomy; mo: month(s), MX StM: mammographically detected stellate mass; n=: data available for n patients, PCT: primary chemotherapy; Pts.: patients; SLNB: sentinel lymph node biopsy; SPM: solitary palpable mass; RLNR: regional lymph node recurrence; RT: radiotherapy; VAB: vacuum assisted biopsy; WE: wide excision, y: year(s).

*1 of 19 spindle cell carcinomas

Table S4. Literature review of low grade adenosquamous carcinoma (LGASC) with data on treatment and outcome.

Reference	# of cases	Age	Presentation	Surgical treatment	LN status	Immuno-phenotype	Adjuvant treatment	Outcome
Yang [95]	1	42	1 PM	WE	NS	ER-<br>PR-	1 No	1 NED (FU 1y)
Bataillon [93]	13	28-85	9 PM<br>4 NPM	1 M<br>12 WE	10 pN0<br>3 NS	13 TN	10 RT<br>4 CHT	13 NED<br>mean FU 7.5y<br>(range 3-17y)
Cheng [96]	1	47	NS	1M	1 NS	NS	1 No	1 NED (FU 12y)
Ohashi [97]	1	76	NS	NS	1 pN0	1 TN	1 NS	1 NED (FU 6y)
Senger [98]	2 (bilateral)	68	2 NPM	2 WE + SLNB	2 pN0	2 TN	2 No	2 NED (FU 4y)
Tan [99]	8	29-49	8 PM	5 WE; 2 SLNB<br>3 M; 2 SLNB	4 pN0	5 TN<br>3 NS	8 No	8 NED<br>median FU 3.4y<br>(range 2.3-4.6y)
Bataillon [100]	3	54-81	2 PM<br>1 NPM	3 WE + SLNB	3 pN0	3 TN	3 No	1 NED (FU 3y)<br>2 NED (FU 6y)
Scali [101]	10	30-81	5 PM<br>3 NPM<br>2 Nipple changes	3 M<br>7 WE<br>(7 ALND)	7 pN0	10 TN	7 RT<br>1 CHT<br>2 NS	1 Local recurrence<br>9 NED<br>(FU 2-11.5y; average 6.5y)
Agrawal [102]	1	19	1 PM	1 M	No SLNB or ALND	NS	1 No	1 NED<br>(FU NS)
Ho [103]	4	51-62	2 PM<br>2 NPM	1 WE + ALND<br>2 M + ALND<br>1 WE	3 pN0	4 TN	1 CHT	4 NED (FU NS)
Ferrara [104]	1	57	1 PM	1 WE + ALND	NS	NS	1 RT	1 NED (FU 3 y)
Shizawa [105]	1	47	1 PM	1 M + ALND	1 pN0	NS	1 No	1 NED (FU 1.3 y)
Foschini [106]	3	46-79	3 PM	1 WE + ALND<br>1 M<br>1 M + ALND	3 pN0	NS	1 CHT	3 NED (FU 7-16 months)

Van Hoeven [107]	32	33-88	32 PM	19 EB + 2 ALND<br>13 M + 11 ALND	12 pN0<br>1 pN+	ER-<br>PR-	32 NS	1 DOD<br>1 AWD (Lung metastases)<br>5 Local recurrences<br>25 NED<br>(FU 1-8.3 y)
Rosen [94]	11	42-76	11 PM	8 EB<br>3 M<br>+ 3 ALND	3 pN0	NS	11 NS	4 Local recurrences<br>7 NED<br>(FU 6 months-6.5 y)

ALND: Axillary lymph node dissection; AWD: Alive with disease; CHT: Chemotherapy ; DOD: Died of disease; EB: Excisional biopsy; ER: oestrogen receptor; FU: Follow-up; M: Mastectomy; NED: No evidence of disease; NPM: Non palpable mass; NS: Not specified; PR: Progesterone receptor; PM: Palpable mass; RT: Radiotherapy; SLNB: Sentinel lymph node biopsy; TN: Triple negative; WE: Wide excision.

Table S5. Summary of reported cases of low or intermediate grade mucoepidermoid carcinoma of the breast.

No.	1st Author [ref.]	Age (years)	Site	Size (cm)	Grade	Surgical Approach	Lymph node metastasis	Distant Metastasis	Adjuvant treatments	Follow-up, (months)	Status
1	Ye [108]	42	R	2.6	LG	MRM	NA	No	CT unknown	12	NED
2	Yan [113]	60	R	1.9	LG	lumpectomy	NA	No	NA	60	NED
3	Burghel [116]	73	L	NA	LG	NA	0/2	No	No	NA	NA
4	Sherwell-Cabello. [117]	86	L	6	LG	MRM	NA	No	No	3	NED
5	Cheng et al. [118]	39	R	1.5	LG	MRM	3/18	No	NA	156	NED
6		49	L	1.5	LG	MRM	0/17	No	NA	41	NED
7		66	L	1.3	LG	Mastectomy + SLD	0/6 (SLD)	No	NA	9	NED
8		61	L	3	LG	Mastectomy + SLD	0/3 (SLD)	No	NA	4	NED

No.	1st Author [ref.]	Age (years)	Site	Size (cm)	Grade	Surgical Approach	Lymph node metastasis	Distant Metastasis	Adjuvant treatments	Follow-up, (months)	Status
9	Fujino [119]	71	R	1.7	IG	Mastectomy + SLD	0/NA	No	NA	NA	NA
10	Camelo-Piragua [111]	49	R	4	IG	MRM	1/3	No	CT (TAC)	8	NED
11	Hornychova [120]	30	L	8	LG	MRM	0/NA	No	CT	60	NED
12	Horii et al. [121]	54	L	2.5	LG	Mastectomy + LND	0/NA	No	ET (ER+PR-HER2-)	36	NED
13	Di Tommaso et al. [109]	80	L	0.5	LG	Excision	NA	No	NA	5	NED
14		29	L	0.8	LG	Excision	NA	No	NA	90	NED
15		54	L	1.5	LG	Quadrantectomy + LND	NA	No	NA	13	NED
16		55	L	1.1	IG	Quadrantectomy + LND	NA	No	NA	3	NED
17	Tjalma [122]	58	R	1.5	LG>HG	RM	1/17	Yes (HG)	RT and tamoxifen (ER-PR-)	156	NED after recurrences (16 mo; RT and excision) and (32 mo; thoracic wall resection)
17	Fisher et al. [123]	65	R	2	LG	Lumpectomy	NA	No	No	60	NED
18		71	L	2	LG	MRM	0/19	No	No	48	NED
19		57	R	2.5	LG	MRM	0/11	No	No	120	NED

No.	1st Author [ref.]	Age (years)	Site	Size (cm)	Grade	Surgical Approach	Lymph node metastasis	Distant Metastasis	Adjuvant treatments	Follow-up, (months)	Status
20		49	R	3.7	LG	RM	0/13	No	No	108	NED
21		60	L	4	LG	SM	NA	No	No	48	NED
22	Patchefsky [115]	66	R	1.3	LG	RM	0/20	No	NA	94	DOD
23		70	R	5	LG	Quadrantectomy	NA	No	NA	10	NED

L: left, R: right; IG: intermediate grade, LG: low grade; (M)RM: (modified) radical mastectomy, LND: lymph node dissection, SM: simple mastectomy; DOD: dead of disease NED: no evidence of disease.

Table S6. Summary of reported cases of secretory carcinoma of the breast.

No of pts	Ref	Age	Size mm	Nodal status	Surgery type	Adjuvant therapy	Follow up	Recurrence, metastasis	Comments
7	McDivitt 1966 [125]	9 (mean)<br>3-15 (range)	10-25 (range)	pN0 (all)	4 excisions (1 followed by re-excision, then M)<br>3 M	No	Median 10y<br>Range (2-15 y)	1 local at 2y after excision, and local again 6y later; NED after 2y	
83	Horowitz 2012 [138]	53 (median); 11-86 (range)	23.6 (mean)	29 (35%) pN+	39 (47%) BCS	35 (42%) RT; no data on systemic therapy	Median 70 mo	5-y CSS: 94%; 10-y CSS: 91%	1983-2007 SEER; includes 2 male pts
3	Lee 2014 [140]	84<br>62<br>23	13<br>6<br>8	pN0 (all 3)	BCS (all 3)	No<br>ET<br>CT	61 mo<br>21 mo<br>14 mo	No<br>No<br>No	 ER+ S100-
246	Jacob 2016 [139]	56.4 (mean)	19.9 (median)	66/206 (32%) pN+	60.4% BCS	90/235 (38.3%) CT; 59/87 (67.8%) ET	>12 y (median not reached)	17 deaths	1998-2011 NCDB; 64% ER+
1	Shui 2017 [132]	53	22	pN+ (1/25)	NA	No	25 mo	No	papillary
1	Shukla 2017 [141]	8	NA	NA	BCS, re-BCS, M	No→CT (5 times), larotrectinib	6 y	1st recurrence at 1y, followed by 4 others, with lung metastases	Successful palliative treatment with TRK inhibitor
1	Ghilli 2018 [127]	5.5	8	pN0	excision	No	48 mo	No	Male (includes review of 32 previously published MBC cases)

1	Sheshe 2018 [142]	20	40	cN0	excision	No	2 mo	No (LFU)	male; pedunculated ulcerated mass contact bleeding
1	Benabu 2018 [143]	70	20	pN0	M	RT	4 y	No	
83*	Garlick 2018 [144]	3-79 (range)	20 (median, n=67); 8-125 (range)	26/52 (50%) pN0	26 BCS<br>57 M	51/75 No; 20/75 CT (6/75 RT, too); 4/75 RT	2,5 y (median; n=60)	56/61 No;<br>4/61 DOD, 1/61 AWD,	8/44 (18%) ER+;<br>42 males
1	Pohlodek 2019 [145]	52	15	pN0	BCS	RT	22 mo	No	
14	Hoda 2019 [130]	55.5 (median)<br>8-81 (range)	16 (median)<br>8-45 (range)	pN+ (2 pts)	10 BCS<br>4 M	4 RT<br>6 CT	89 mo (mean)<br>21-212 mo (range)	2 pts with axillary met<br>2 pts with distant metastasis	2 males<br>6/10 ER+, 3/10 PR+, too
1	Yang 2019 [131]	38	40	pN0	M	No	13 mo	No	in situ
44	Li 2019 [124]	48 (median)<br>4-76 (range)	35 (mean)<br>15-100 (range)	29/44 pN0	2 BCS<br>41 M<br>1?	CT 40 pts<br>RT 11 pts	93.4 mo (median); 10-182 mo (range)	5/44 (11%) DOD; 6/44 (14%) metastasis or recurrence	21/44 (48%) ER+; 16/44 HER2+; 17 TNBC; only 30/44 positive with FISH for the ETV6-NTRK3 rearrangement; 3 males
1	Novochadlo Klüppel	9	35	pN0	M	No	16 mo	No	male

	2020 [146]								
1	Gohara 2020 [147]	6	12	cN0	BCS (excision)	No	NA	No	ER+ PR- HER2-
1	Xu 2020 [129]	61	30	pN0	M	CT + ET	14 mo	Metastasis (lung, bones, paraspinal soft tissues) 8 mo; DOD 14 mo	Sarcomatous dedifferentiation
1	Altundag 2020 [148]	56	40	pN0	M	CT	NA	No	Multicentric tumour

* without cases by McDivitt and Stewart;

BCS: breast conserving surgery, cN0: clinically node-negative, CT: chemotherapy, ER: oestrogen receptor, ET: endocrine therapy, FISH: fluorescent in situ hybridisation, HER2: human epidermal growth factor receptor-2, M: mastectomy, mo: month(s), NA: not available / not applicable, NCDB: national Cancer Database, pN0: pathologically node-negative, pN+, pathologically node-positive, PR: progesterone receptor, pts: patients, RT: radiotherapy, SEER: Survival Epidemiology and End Results, y: year(s)
